# Enhancing Nutritional and Health Benefits of Wheat Bran Through Bifunctional LAB Screening and BCAA-Enriched Fermentation

**DOI:** 10.3390/foods15142555

**Published:** 2026-07-20

**Authors:** Byung Hoon Lee, Sun Ok Han, Jun Seok Hong, Seung Jo Jeong, Ji Youn Hong, Young Jun Kim

**Affiliations:** 1 Food R&D Institute, Samyang Foods Inc., 159 Toegye-ro, Seoul 04553, Republic of Korea; 2 Department of Food Regulatory Science, Korea University, 2511 Sejong-ro, Sejong, 30019, Republic of Korea; 3 Department of Food & Biotechnology, Korea University, 2511 Sejong-ro, Sejong 30019, Republic of Korea

**Keywords:** lactic acid bacteria, wheat bran, fermentation, branched-chain amino acids, proteolytic activity, nutritional bioavailability

## Abstract

Lactic acid bacteria (LAB)-mediated fermentation has been widely explored as a strategy to enhance the nutritional functionality of cereal processing by-products. In this study, 50 LAB strains previously isolated from Korean traditional fermented foods and obtained from the National Agrobiodiversity Center (KACC, Jeonju-si, Republic of Korea) were systematically screened for bifunctional carbohydrate and protein degradation capacities, and their potential to improve the nutritional functionality of wheat bran was evaluated. Paper disc assays revealed substantial inter-strain variability, with clear zone diameters ranging from 12.35 to 29.52 mm for carbohydrate degradation and 11.61 to 25.45 mm for protein degradation. Ten strains exceeding both upper-quartile enzymatic degradation cutoff thresholds (≥25.25 mm for carbohydrate degradation and ≥17.98 mm for protein degradation, respectively) were putatively identified as *Lactiplantibacillus plantarum* and *Lacticaseibacillus paracasei* based on 16S rRNA gene sequencing (99.73–100% similarity). Substrate-specific fermentation using *L. paracasei* KS 595 across four substrates (brewed soy sauce soybean meal, pea, floury rice, and wheat bran) demonstrated substrate-dependent differences in growth and branched-chain amino acid (BCAA) accumulation, with the highest increase observed in pea fermentation. Strain-specific evaluation during wheat bran fermentation revealed distinct differences in growth kinetics, pH reduction, and BCAA production driven by cell-envelope proteinases and intracellular peptidases, with viable cell counts reaching 9–10 log CFU/mL after 48 h. Biogenic amine analysis indicated that histamine levels remained below commonly accepted safety limits in all strains, while *L. paracasei* KS 543 showed no detectable tyramine or histamine. Although the potential improvement in plant protein bioavailability was indirectly inferred through the free BCAA enrichment rather than directly measured *in vivo*, these results support a systematic screening approach for selecting LAB strains capable of producing BCAA-enriched fermented wheat bran, with potential implications for improving plant protein bioavailability and addressing nutritional needs in aging and active populations.

## 1. Introduction

Cereal-based foods constitute the nutritional foundation of human nutrition worldwide given their substantial contribution to dietary energy and protein intake across diverse populations; however, their nutritional functionality is often limited by processing-related constraints and the inherent composition of raw materials [1,2]. Conventional processing conditions may restrict the incorporation or accessibility of nutritionally beneficial components, highlighting the need for alternative strategies to enhance substrate functionality at the raw material level [1]. Improving the nutritional quality of cereal-based substrates requires fermentation strategies that are both effective in modifying composition and compatible with existing food processing conditions [2]. Lactic acid bacteria (LAB) mediated fermentation has been reported to contribute to the modification of the nutritional and structural matrices of cereal substrates, offering an approach for upstream functional enhancement [3,4].

Fermented foods are well-established sources of LAB with diverse enzymatic and metabolic activities [5]. LAB have been widely investigated for their roles in protein and carbohydrate degradation, as well as their capacity to modulate the nutritional and sensory characteristics of fermented substrates [6]. In particular, LAB originating from diverse ecological niches represent valuable resources for obtaining strain-specific fermentation functionalities [7,8]. Despite extensive research on well-characterized strains, no study has systematically integrated bifunctional enzymatic screening, multi-substrate fermentation evaluation, and safety profiling within a single experimental framework for LAB isolates derived from Korean meju. Furthermore, the relationship between carbohydrate degradation capacity and fermentative competitiveness in protein-rich substrates such as wheat bran remains insufficiently characterized at the strain level.

LAB-mediated fermentation has emerged as a robust strategy for the biotransformation of raw materials, facilitating the enhancement of substrate functionality [5,6,9]. Leveraging these metabolic advantages, cereal processing by-products represent promising substrates for fermentation-based valorization [9,10,11]. Particularly, wheat is one of the most widely cultivated cereal crops globally, generating substantial quantities of wheat bran during the milling process [12]. Although this by-product is inherently rich in dietary fiber, protein, and bioactive compounds, its food applications remain heavily restricted due to the high concentration of insoluble dietary fiber [11], the presence of anti-nutritional factors such as phytates [13], and the structural constraints of the complex plant cell wall matrix [11], which collectively impede nutrient accessibility and diminish sensory quality [14]. Lactic acid fermentation has been reported to partially mitigate these issues by structural modification of the cereal matrix, thereby potentially improving nutrient bioavailability and sensory-related attributes [5,6,14]. Consequently, wheat bran fermentation offers a sustainable pathway for converting underutilized milling by-products into nutrient-dense, value-added ingredients [15,16]. In addition to these sustainability-related merits, LAB-mediated fermentation of cereal by-products holds significant nutritional relevance in the context of growing plant-based dietary trends, where improving protein quality and amino acid bioavailability of plant-derived substrates has emerged as a public health priority [3,17]. In particular, branched-chain amino acids (BCAAs) including leucine, isoleucine, and valine are recognized for their roles in stimulating muscle protein synthesis via the mechanistic target of rapamycin (mTOR) signaling pathway and in counteracting anabolic resistance associated with aging and sarcopenia [18,19]. Therefore, strategies that enhance BCAA content in plant-based fermented substrates may contribute to both the nutritional functionality and health-promoting potential of cereal-derived food ingredients.

The efficacy of wheat bran fermentation in this regard is largely driven by the proteolytic system of LAB. This process is initiated by extracellular proteinases, including cell-envelope proteinases (CEPs) in strains that harbor functional *CEP* genes, which catalyze the extracellular hydrolysis of macromolecular proteins into oligopeptides, which are subsequently internalized and degraded by intracellular peptidases into free amino acids [20]. In cereal-derived substrates, major protein fractions, specifically albumins and globulins, serve as primary substrates for microbial proteolysis, leading to the liberation of essential branched-chain amino acids (BCAAs). Recent studies indicate that LAB strains with potent proteolytic activity can significantly elevate concentrations of valine, leucine, and isoleucine in fermented plant-based matrices [21,22]. However, as proteolytic capacity is highly strain-dependent, a systematic evaluation of diverse isolates is essential to identify optimal candidates for effective substrate valorization.

Therefore, the present study screened 50 LAB strains previously isolated from Korean traditional fermented foods and obtained from the National Agrobiodiversity Center (KACC, Korea) for carbohydrate- and/or protein-degrading activities. Their fermentation performance was evaluated across four distinct substrates, with a particular focus on wheat bran. The effects of selected strains on bacterial growth kinetics, pH dynamics, BCAA profiles, and biogenic amine accumulation were characterized to identify strains suitable for enhancing the functional value of cereal processing by-products. We hypothesized that LAB strains possessing bifunctional amylolytic and proteolytic activities would more effectively overcome the structural matrix constraints of wheat bran, thereby demonstrating superior growth competitiveness and greater BCAA liberation compared to strains exhibiting only proteolytic activity.

## 2. Materials and Methods

### 2.1. Substrates and LAB

Brewed soy sauce soybean meal (protein 22.47%, carbohydrate 21.60%), pea (carbohydrate 71.01%, protein 22.60%), wheat bran (protein 15.48%, carbohydrate 62.77%), and floury rice (protein 8.16%, carbohydrate 81.12%) were provided as dried materials by Samyang Food R&D Institute (Samyang Foods Inc., Seoul, Republic of Korea). The brewed soy sauce soybean meal was further dried using a hot air dryer to a moisture content below 5% prior to use.

A total of 50 LAB strains were obtained from the National Agrobiodiversity Center at the National Institute of Agricultural Sciences. These strains were originally isolated from Korean traditional fermented foods (meju) and related biological sources. For activation, strains were subcultured twice in MRS broth (Difco, Franklin Lakes, NJ, USA) at 37 °C for 48 h under static conditions. Activated cultures were streaked onto BCP agar plates (Mbcell, Seoul, Korea, Cat. No. MB-P1601) and incubated at 37 °C for 48 h. Yellow colonies, indicative of acid production, were selected and subcultured at least twice in MRS broth to obtain pure isolates. Viable cell counts were confirmed to exceed 10^9^ CFU/mL by plating on BCP agar. Purity of all isolates was confirmed by colony morphology assessment on BCP agar and Gram staining prior to downstream analysis. Although these strains were obtained from KACC, some strains were not yet officially registered in the repository at the time of this study. Therefore, independent 16S rRNA gene sequencing was performed to confirm strain identity under our specific laboratory conditions and to obtain GenBank accession numbers for independent strain traceability.

### 2.2. Evaluation of Protein and Carbohydrate Degradation Capacity

Although LAB generally lack cellulolytic and ligninolytic activities, amylolytic activity was selected as the representative indicator of carbohydrate degradation capacity, as the residual starch fraction of wheat bran serves as the primary accessible carbon source for initial microbial growth [6,20]. The proteolytic and amylolytic activities were evaluated using a modified paper disc diffusion assay [23,24]. For proteolytic activity, PCA medium (Mbcell, Seoul, Korea, Cat. No. MB-P1040) supplemented with 1.6% (*w*/*v*) skim milk (Difco, Detroit, MI, USA, Cat. No. 232100) was used. For amylolytic activity, MRS agar containing 1% (*w*/*v*) soluble starch (Difco, Franklin Lakes, NJ, USA, Cat. No. 217820) was prepared. Bacterial cultures (30 μL) were applied to sterile paper discs placed on the respective media and incubated at 37 °C for 48 h. Proteolytic activity was determined by the formation of clear zones, whereas amylolytic activity was visualized by flooding the plates with iodine solution (Taejin Sangsa, Seoul, Korea) followed by clear zone observation. Clear zone diameters were measured using ImageJ software (National Institutes of Health, Bethesda, MD, USA). The upper quartile thresholds (≥25.25 mm for carbohydrate degradation and ≥17.98 mm for protein degradation) were used as selection thresholds. Strains satisfying both criteria were designated as bifunctional candidates.

### 2.3. 16S rRNA Gene Sequencing

Selected strains were identified by 16S rRNA gene sequencing. Polymerase chain reaction (PCR) amplification was performed using primers 27F (5′-AGAGTTTGATCCTGGCTCAG-3′) and 1492R (5′-GGTTACCTTGTTACGACTT-3′) with a 2X PCR mixture (Solgent, Daejeon, Republic of Korea). The PCR conditions were as follows: initial denaturation at 95 °C for 10 min; 28 cycles of 95 °C for 45 s, 55 °C for 1 min, and 72 °C for 90 s; and a final extension at 72 °C for 10 min. PCR products were verified by 1% agarose gel electrophoresis and subjected to Sanger sequencing by Macrogen (Seoul, Korea). Forward and reverse sequences were assembled to generate consensus sequences, and sequence similarity was determined using BLASTn searches against the NCBI 16S ribosomal RNA sequences database, with species-level identification assigned at a sequence identity threshold of ≥99%. For phylogenetic analysis, reference 16S rRNA gene sequences were retrieved from the NCBI 16S ribosomal RNA database, aligned using ClustalW (Conway Institute UCD, Dublin, Ireland), and used to construct a phylogenetic tree with the PHYLIP package (University of Washington, Seattle, WA, USA).

### 2.4. Evaluation of Substrate-Specific Fermentation Characteristics

Fermentation experiments were conducted with minor modifications based on Mao et al. [25]. Although solid-state fermentation (SSF) more closely approximates industrial wheat bran processing conditions, submerged fermentation was adopted in this study to ensure uniform substrate-microbe mixing, precise temporal sampling, and consistent control of fermentation variables across the multi-strain and multi-substrate experimental setups. These aspects serve as essential prerequisites for high-throughput comparative strain screening and evaluation. *Lacticaseibacillus paracasei* KS 595, which exhibited the highest degradation capacity and 100% sequence identity to reference strains, was selected. Substrates were sterilized at 121 °C for 15 min prior to fermentation, and all experiments were conducted under controlled conditions to ensure reproducibility across substrates. Each substrate (200 g) was mixed with 1 L of sterile water (1:5, *w*/*v*). LAB cultures (approximately 10^9^ CFU/mL) were inoculated at 1% (*v*/*w*) and incubated at 37 °C for 48 h under shaking conditions. Samples were collected at 0, 2, 4, 8, 12, 16, 20, 24, 36, and 48 h for analysis [21,26]. For analysis, 25 g of sample was diluted (1:1) with 0.85% NaCl solution. Viable LAB counts were determined using BCP agar, and pH was measured using a pH meter (STARA2116, Thermo Fisher Scientific, Waltham, MA, USA).

### 2.5. Free Amino Acid Analysis During Substrate Fermentation

Samples (20 mL) were centrifuged at 10,000× *g* for 10 min at 4 °C (Model A32020(2) Labogene, Gimpo, Republic of Korea), filtered through a 0.2 μm PVDF membrane (Whatman, GE Healthcare, Buckinghamshire, UK), and stored at −80 °C. Samples were subsequently freeze-dried prior to analysis. Freeze-dried samples were diluted (1:10) in ultrapure 0.1% formic acid. A 100 μL aliquot was mixed with 900 μL methanol (0.1% formic acid), vortexed, and centrifuged (10,000× *g*, 10 min, 4 °C). The supernatant was used for analysis. LC-MS/MS analysis was performed using a Waters ACQUITY UPLC system coupled with a Xevo TQ-S tandem mass spectrometer (Waters Corporation, Milford, MA, USA). Separation was achieved using an Intrada Amino Acid column (50 mm × 2 mm, 3 μm). Mobile phases consisted of (A) acetonitrile containing 100 mM ammonium formate (20:80, *v*/*v*) and (B) acetonitrile:THF:25 mM ammonium formate:formic acid (9:75:16:0.3, *v*/*v*/*v*/*v*). Gradient elution was applied as follows: 0–3 min, 100% B; 3–6.5 min, 100–83% B; 6.5–10 min, 83–0% B; 10–12 min, 0–100% B; 12–17 min, 100% B. The flow rate was maintained at 0.4 mL/min. Multiple reaction monitoring (MRM) conditions were optimized in positive electrospray ionization mode (ESI+). The key MS/MS source parameters were maintained as follows: capillary voltage, 3.0 kV; source temperature, 150 °C; desolvation temperature, 450 °C; and desolvation gas flow, 800 L/h. In addition, compound-specific MRM transitions and parameters were as follows. For example, valine showed a transition of 118.1 → 72.05 *m*/*z* (cone voltage, 26 V; collision energy, 12 eV; retention time, 2.21 min). Notably, the structural isomers leucine and isoleucine, which share an identical MRM transition (132.1 → 86.15 *m*/*z*; cone voltage, 26 V; collision energy, 8 eV), were successfully resolved chromatographically with distinct retention times of 1.64 min and 1.80 min, respectively. Quantification was performed using a four-point calibration curve with concentrations of 10, 20, 50, and 100 nmol/mL for each amino acid processed through the instrument’s dedicated data analysis software. The limits of quantification (LOQ) for the amino acids ranged from 10 to 50 nmol/mL. The complete MRM transitions, cone voltages, collision energies, and LOQs are presented in Table S2.

### 2.6. Evaluation of Strain-Specific Wheat Bran Fermentation

Eight LAB strains were selected for wheat bran fermentation: four strains meeting both degradation criteria (KS 543, KS 548, KS 550, and KS 568) and four strains meeting only the proteolytic criterion (KS 545, KS 565, KS 574, and KS 597). Wheat bran was sterilized at 130 °C for 30 min to eliminate heat-resistant endospores, as identified in preliminary tests. This condition was applied in accordance with established protocols for thermophilic spore-former decontamination in cereal matrices [27]. For fermentation, 200 g of substrate was mixed with 1 L of sterile water (5-fold dilution). LAB cultures (approximately 10^9^ CFU/mL) were inoculated at a final concentration of 1% (*v*/*w*) and incubated at 37 °C for 48 h using a shaking incubator. Samples were collected at 0, 2, 4, 8, 12, 16, 20, 24, 36, and 48 h for analysis. For sampling, 25 g of fermentation product was diluted (1:1) with 0.85% NaCl solution. Total viable LAB counts were determined by plating on BCP medium, and pH was monitored using a pH meter (STARA2116, Thermo Fisher Scientific, Waltham, MA, USA).

### 2.7. Quantitative Analysis of Amino Acids Using LC-MS/MS

Amino acid quantification in this section was performed using a different analytical platform from that used in Section 2.5, reflecting instrument availability during distinct experimental periods. To ensure quantitative comparability, both methods were independently validated using identical amino acid standard mixtures, and calibration curves for the BCAAs (valine, leucine, and isoleucine) yielded equivalent results within ±5% analytical deviation. Free amino acid analysis was performed on fermentation samples collected at each time point. Sample preparation, including centrifugation, filtration, storage, and freeze-drying, was conducted as described in Section 2.5. Amino acid extraction and chromatographic analysis were performed according to Huang et al. [28] with minor modifications. Freeze-dried samples were reconstituted in 0.1 M HCl (1:100, *w*/*v*), vortexed, and sonicated for 15 min. After centrifugation at 7000× *g* for 10 min at 4 °C, 100 μL of the supernatant was diluted with 400 μL of mobile phase B and filtered through a 0.2 μm PTFE syringe filter prior to analysis [29].

Chromatographic separation was performed using a Vanquish HPLC system coupled with a TSQ Altis Plus triple quadrupole mass spectrometer (Thermo Fisher Scientific) equipped with an Agilent InfinityLab Poroshell 120 HILIC-Z column, Agilent Technologies, Santa Clara, CA, USA (2.1 mm × 150 mm, 2.7 μm). Mobile phase A consisted of 10% 200 mM ammonium formate (pH 3 with formic acid), and mobile phase B consisted of 10% 200 mM ammonium formate (pH 3 with formic acid) in acetonitrile. Gradient elution was applied as follows: 0 min, 100% B; 7 min, 89% B; 10 min, 88% B; 16 min, 75% B; and 16.5–20 min, 100% B. The flow rate was 0.4 mL/min, and the column temperature was maintained at 30 °C. The mass spectrometer was operated in ESI-positive mode, and SRM transitions for amino acids were optimized prior to analysis using the TSQ Tune Application software (Thermo Fisher Scientific, Waltham, MA, USA) for each of the 14 target amino acids. Detailed SRM transition parameters for all target amino acids are listed in Supplementary Table S3.

Quantification was performed using external calibration curves constructed from mixed amino acid standards at concentrations ranging from 10 to 200 nmol/mL. Standard solutions were prepared by diluting stock solutions with mobile phase B. Calibration curves were generated for each amino acid by plotting peak area against concentration, and amino acid concentrations in the samples were calculated using the corresponding regression equations. Excellent linearity was obtained for all amino acids over the calibration range (R^2^ >0.99).

### 2.8. Assessment of Biogenic Amine Production

Biogenic amine analysis was performed according to Park et al. [30] with minor modifications. MRS broth was supplemented with 0.5% (*w*/*v*) each of L-histidine monohydrochloride monohydrate, L-tyrosine disodium salt hydrate, L-ornithine monohydrochloride, and L-lysine monohydrochloride, along with 0.0005% (*w*/*v*) pyridoxal-HCl as a coenzyme. The final pH of the medium was adjusted to 5.8 using 1 M HCl prior to sterilization. The prepared medium was incubated with the respective LAB cultures and incubated at 37 °C for 48 h under static conditions.

After incubation, samples were filtered through a 0.2 μm membrane filter, and biogenic amines were extracted using 0.4 M perchloric acid at 4 °C for 2 h, followed by centrifugation. The supernatant was re-filtered and used for analysis.

For derivatization, 1 mL of sample was mixed with 200 μL of 2 M NaOH and 300 μL of saturated sodium bicarbonate, followed by the addition of 2 mL of dansyl chloride solution (10 mg/mL in acetone). The mixture was reacted at 40 °C for 45 min. Subsequently, 100 μL of 25% ammonium hydroxide was added and reacted at 25 °C for 30 min to remove residual dansyl chloride. The volume was adjusted to 5 mL with acetonitrile, followed by centrifugation. For quantitative analysis, external standards (Sigma-Aldrich, Saint Louis, MO, USA; purity ≥ 98%) including tryptamine, β-phenylethylamine, putrescine, cadaverine, histamine, tyramine, spermidine, and spermine were used to construct calibration curves (0–1000 ppm).

Analysis was performed using a Waters HPLC system equipped with a UV detector (254 nm) and a Nova-Pak C18 column (150 mm × 4.6 mm, 4 μm, Waters Waters Corporation, Milford, MA, USA)). Mobile phase A consisted of 0.1 M ammonium acetate in double-distilled water (DDW), and mobile phase B consisted of acetonitrile. Gradient elution was applied as follows: 0 min, 25% B; 5 min, 45% B; 18 min, 60% B; 25 min, 75% B; and 30 min, 25% B. The flow rate was maintained at 1.0 mL/min, the injection volume was 10 μL and the column temperature was maintained at 40 °C.

### 2.9. Statistical Analysis

All statistical analyses were performed using Prism 8.0.2 software (GraphPad Software Inc., La Jolla, CA, USA), and results are expressed as means ± standard deviation (SD). Prior to statistical testing, the normality of data distribution and the homogeneity of variances were verified using the Shapiro–Wilk test and Levene’s test, respectively. Overall differences between groups were analyzed using one-way analysis of variance (ANOVA). Tukey’s multiple comparisons test was applied as a post hoc analysis to evaluate pairwise differences among all time points, including comparisons against the initial time point (0 h) and between consecutive time points. Statistical significance was indicated as follows: * *p* < 0.05, ** *p* < 0.01, *** *p* < 0.001 compared to the initial time point (0 h); # *p* < 0.05, ## *p* < 0.01, and ### *p* < 0.001 compared to the previous time point.

## 3. Results

### 3.1. Carbohydrate and Protein Degradation Capacity of LAB Strains

Carbohydrate and protein degradation capacities of 50 LAB strains were evaluated by measuring clear zone diameters using a paper disc diffusion assay (Table S4; Figure S1). Clear zone diameters ranged from 12.35 ± 7.13 mm (KS 552) to 29.52 ± 2.83 mm (KS 546) for carbohydrate degradation and from 11.61 ± 0.66 mm (KS 191) to 25.45 ± 0.24 mm (KS 569) for protein degradation, demonstrating substantial strain-dependent variability.

**Figure 1:**
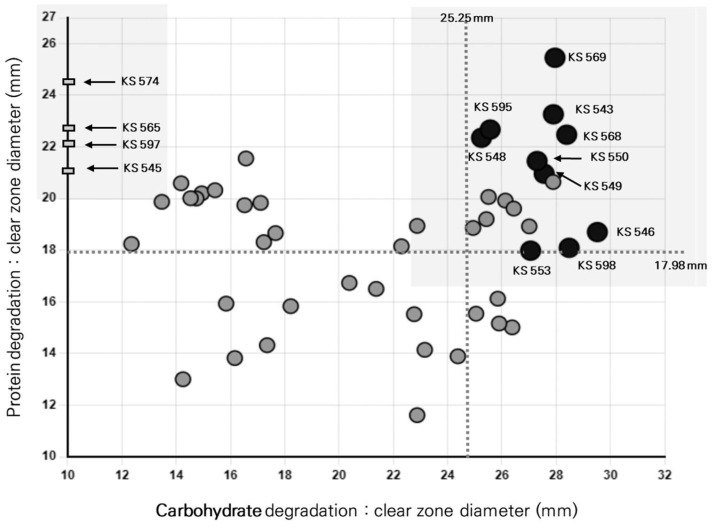
Distribution of carbohydrate and protein degradation activities of 50 lactic acid bacteria strains based on clear zone diameter. Filled circles (●, black) indicate the ten selected strains that simultaneously exceeded the threshold values for both carbohydrate degradation (≥25.25 mm) and protein degradation (≥17.98 mm). Grey circles (●, grey) represent the remaining strains. The open squares on the left indicate strains with superior protein degradation activity only, without detectable carbohydrate degradation activity (KS 545, KS 565, KS 574, and KS 597). Dashed lines indicate the respective cutoff thresholds.

To identify strains with superior bifunctional degradation capacity suitable for efficient fermentation starter development, stringent threshold values were established at the upper quartile of each distribution: ≥25.25 mm for carbohydrate degradation and ≥17.98 mm for protein degradation. Ten strains simultaneously exceeded both thresholds and were designated as selected strains (Figure 1, filled circles). An additional four strains (KS 545, KS 565, KS 574, and KS 597) exhibited protein degradation activity above the threshold but showed no detectable carbohydrate degradation activity (Figure 1, open squares) and were included for comparative evaluation in subsequent fermentation experiments.

### 3.2. Molecular Identification of Selected LAB Strains by 16S rRNA Gene Sequencing

Ten strains were selected for fermentation evaluation; six meeting both degradation thresholds and four meeting the protein degradation criterion only were subjected to 16S rRNA gene sequencing and BLAST (National Center for Biotechnology Information, Bethesda, MD, USA) analysis against the GenBank database (Table 1). All ten strains were identified as either *Lacticaseibacillus paracasei* or *Lactiplantibacillus plantarum*, with sequence identities ranging from 99.73% to 100%. Among the five dually selected strains, KS 543, KS 548, and KS 595 were identified as *L. paracasei* from 99.8% to 100% sequence identity to the reference sequence AP012541.1, while KS 550 and KS 568 were identified as *L. plantarum*. Among the four protein-only strains, KS 545 and KS 597 were identified as *L. paracasei*, and KS 565 and KS 574 as *L. plantarum*. All strains were originally isolated from meju, a traditional Korean fermented soybean product. Of the ten identified strains, KS 569 (*L. plantarum*, 99.86%) exhibited insufficient fermentative growth under the screening conditions applied and was excluded from subsequent fermentation experiments. KS 595 was selected as the representative strain for substrate-specific fermentation comparison (Section 3.3), while the remaining eight strains were used for strain-specific wheat bran fermentation evaluation (Section 3.5 and Section 3.6).

### 3.3. Substrate-Specific Fermentation Characteristics of L. paracasei KS 595

**Figure 2:**
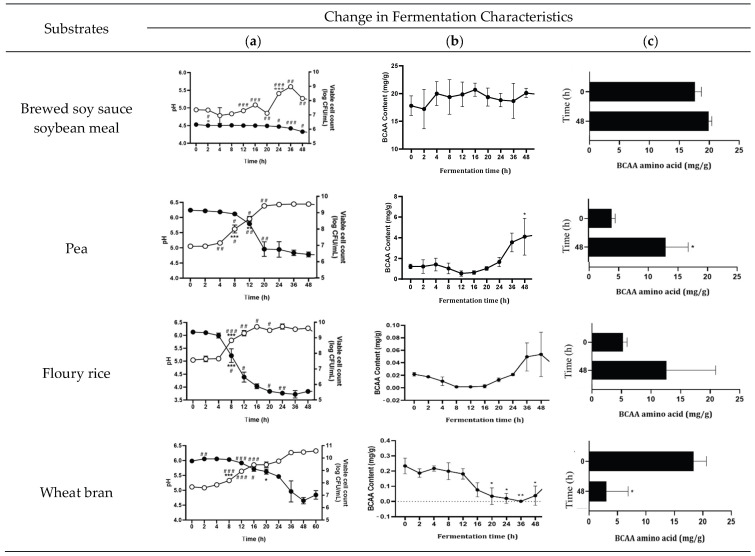
Changes in pH, viable cell counts, and BCAA amino acid contents during fermentation of four substrates by *Lacticaseibacillus paracasei* KS 595 over 48 h. (**a**) pH and viable cell counts, (**b**) time-course changes in total BCAA amino acid contents, and (**c**) comparison of total BCAA amino acid contents between 0 h and 48 h. Data are expressed as mean ± SD (*n* = 3). Open circles (○) and closed circles (●) represent pH and viable cell counts, respectively. The dotted horizontal line in the wheat bran panel indicates zero reference. Asterisks indicate significant differences compared to 0 h (* *p* < 0.05, ** *p* < 0.01, *** *p* < 0.001). Hash symbols indicate significant differences compared to the previous time point (# *p* < 0.05, ## *p* < 0.01, ### *p* < 0.001).

To evaluate substrate-specific fermentation performance, *L. paracasei* KS 595, which exhibited 100% sequence identity to the reference strain, simultaneously fulfilled both degradation thresholds, and demonstrated favorable growth performance across preliminary screening conditions, was selected as a representative strain. Changes in pH, viable cell counts, and BCAA amino acid contents were monitored over 48 h across four substrates: brewed soy sauce soybean meal, pea, floury rice, and wheat bran (Figure 2).

In the case of brewed soy sauce soybean meal, viable cell counts remained relatively stable throughout the fermentation period without a pronounced growth phase (no statistically significant change observed between any time points, *p* > 0.05), and pH showed no significant decrease. Correspondingly, BCAA amino acid contents showed no statistically significant change between 0 h and 48 h.

In pea fermentation, viable cell counts increased rapidly after 8 h, reaching approximately 9–10 log CFU/mL by 12 h, accompanied by a concurrent and significant pH decrease. Pea contained a relatively high protein content (22.6 g/100 g; Table S1), and BCAA amino acid contents increased progressively and showed a significant increase at 48 h compared to 0 h (*p* < 0.05).

For floury rice, a clear increase in viable cell counts was observed after 8 h, and pH decreased significantly as fermentation progressed. Floury rice contained a high carbohydrate content (81.12 g/100 g) but a relatively low protein content (8.16 g/100 g; Table S1).

In wheat bran fermentation, gradual bacterial growth was observed after 8 h, followed by a mild but progressive pH decrease. Although initial BCAA amino acid contents at 0 h were relatively high, values declined during the early to mid-fermentation phase and showed partial recovery in the late stage, resulting in a statistically significant net decrease at 48 h compared to 0 h (*p* < 0.05). Although a statistically significant net decrease in total BCAA content was observed at 48 h during wheat bran fermentation with KS 595, this highlights the critical importance of strain-specific metabolic characteristics in utilizing wheat bran as a fermentation substrate. While wheat bran is rich in protein precursors, certain strains may consume free BCAAs to support cellular growth or metabolic pathways during prolonged fermentation. Subsequent strain-specific evaluation (Section 3.5 and Section 3.6) was therefore conducted to identify optimal strains for BCAA enrichment in wheat bran.

### 3.4. Free Amino Acid Profiles During Substrate-Specific Fermentation

To characterize the amino acid biotransformation capacity of KS 595 across substrates, 20 free amino acids were quantified at each sampling time point. In brewed soy sauce soybean meal fermentation, no statistically significant changes were observed for most amino acids under the osmotic stress conditions imposed by the high-sodium substrate. Gly, Ala, Glu, and Tyr showed gradual increasing trends, while Met and Cys showed decreasing patterns over the fermentation period with no statistical significance (*p* > 0.05).

In pea fermentation, BCAA (Val, Leu, Ile) contents increased significantly during fermentation. Leu increased rapidly after the mid-stage of fermentation, while Val and Ile showed increasing trends in the later stage. Concurrently, Arg and Glu also increased as fermentation progressed. In floury rice fermentation, most amino acids decreased in the early stage but showed increasing trends from the mid- to late-stage, with BCAA contents increasing significantly after 16–20 h. Arg showed a particularly rapid increase during the mid-stage, suggesting preferential arginine release by the protease system of KS 595.

In wheat bran fermentation, most amino acids decreased during the early fermentation stage and subsequently showed increasing trends during the later stage. BCAA amino acids declined until the mid-stage and showed partial recovery thereafter. Overall, the magnitude of BCAA changes between 0 h and 48 h was greatest in pea fermentation, followed by brewed soy sauce soybean meal, wheat bran, and floury rice. Detailed free amino acid profiles for all substrates across the full time course are provided in Table S5.

### 3.5. Strain-Specific Fermentation Characteristics During Wheat Bran Fermentation

**Figure 3:**
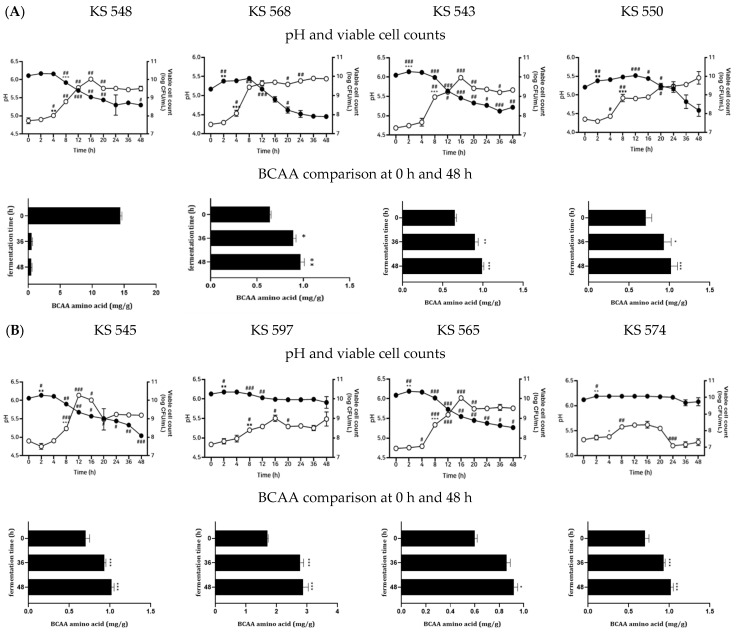
Changes in pH, viable cell counts, and BCAA amino acid contents during wheat bran fermentation over 48 h for strains selected based on degradation activity criteria. (**A**) Strains meeting both carbohydrate and protein degradation thresholds (KS 548, KS 568, KS 543, and KS 550); (**B**) Strains meeting the protein degradation threshold only (KS 545, KS 597, KS 565, and KS 574). Data are expressed as mean ± SD (*n* = 3). Open circles (○) and closed circles (●) represent pH and viable cell counts, respectively. Asterisks indicate significant differences compared to 0 h (* *p* < 0.05, ** *p* < 0.01, *** *p* < 0.001). Hash symbols indicate significant differences compared to the previous time point (# *p* < 0.05, ## *p* < 0.01, ### *p* < 0.001).

Given the pronounced BCAA enrichment observed in wheat bran fermentation and its relevance as a cereal processing by-product, wheat bran was selected for strain-specific comparative analysis. Changes in viable cell counts, pH, and BCAA amino acid contents were assessed for all eight LAB strains over 48 h (Figure 3).

Most strains exhibited increasing viable cell counts after the initial fermentation stage, with the onset of active growth generally occurring after 8 h, though some strains initiated growth as early as 4 h. At the end of fermentation, viable cell counts of approximately 9–10 log CFU/mL were achieved in most strains. Among the dually selected strains (Figure 3A), KS 568 showed the most rapid growth onset and the highest viable cell count at 48 h, while KS 548 exhibited a more gradual growth pattern. Among the protein-only strains (Figure 3B), KS 597 and KS 574 demonstrated notably lower bacterial growth and pH decrease, indicating reduced fermentative competitiveness in wheat bran. Strains meeting both degradation thresholds (KS 543, KS 548, KS 550, KS 568) showed significantly higher viable cell counts and greater pH reduction compared to protein-only strains (KS 545, KS 565, KS 574, KS 597) at 48 h (*p* < 0.05).

### 3.6. BCAA Amino Acid Contents During Strain-Specific Wheat Bran Fermentation

Changes in BCAA contents during wheat bran fermentation showed clear strain-dependent differences (Figure 3, Table S5). At 36 h, BCAA contents increased for most strains, with the exception of KS 548, which showed a transient decrease at 36 h (*p* < 0.05). By 48 h, statistically significant increases in BCAA amino acid contents compared to 0 h were confirmed for KS 568, KS 543, KS 550, KS 545, KS 565, and KS 574 (*p* < 0.05 to *p* < 0.001). Among all strains, KS 597 exhibited the highest absolute BCAA content at 48 h, which should be interpreted alongside its biogenic amine profile discussed in Section 3.7. Detailed free amino acid profiles for all strains across the full time course are provided in Table S5.

### 3.7. Biogenic Amine Production Profiles

**Figure 4:**
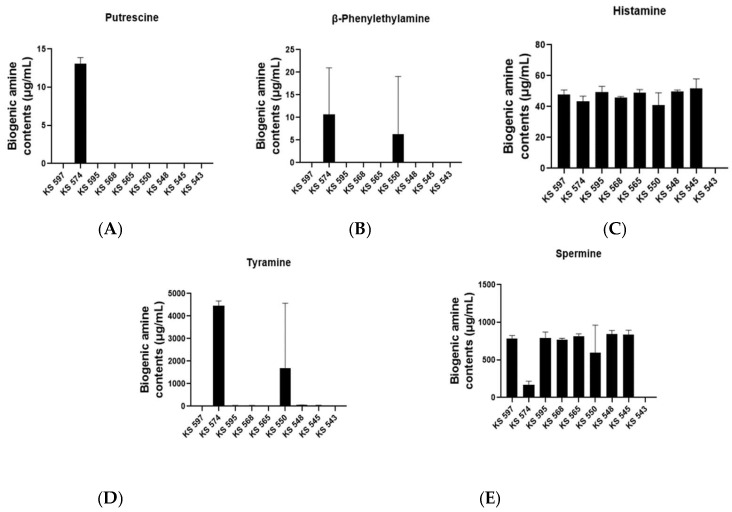
Biogenic amine contents in wheat bran fermented by eight lactic acid bacteria strains. Data are expressed as mean ± SD (*n* = 3). (**A**) Putrescine, (**B**) β-phenylethylamine, (**C**) histamine, (**D**) tyramine, and (**E**) spermine. Tryptamine, cadaverine, and spermidine were not detected (ND) in any of the tested strains.

To evaluate the safety of the selected strains for potential food applications, biogenic amine production was assessed in MRS broth supplemented with amine precursor amino acids (Figure 4). Tryptamine, cadaverine, and spermidine were not detected in any of the tested strains, indicating the absence of the corresponding decarboxylase activities.

β-Phenylethylamine was detected only at trace levels in KS 574 (10 μg/mL) and KS 550 (6 μg/mL), both well below the toxicological safety threshold. Putrescine was similarly absent in most strains, with only KS 574 producing detectable levels (13 μg/mL). Histamine was detected across most strains at concentrations ranging from approximately 40 to 55 μg/mL, all well below the toxicological safety limit of 100 mg/kg generally recommended by regulatory agencies. KS 543 produced no detectable histamine. Spermine was detected across most strains at 500–850 μg/mL, with KS 574 producing comparatively low levels (172 μg/mL).

Tyramine production showed the greatest inter-strain variation and represented the most significant safety concern. KS 574 produced markedly elevated tyramine (~4500 μg/mL) and KS 550 also exceeded the commonly referenced safety range (100–800 μg/mL) with approximately 1700 μg/mL. This finding highlights the importance of incorporating biogenic amine profiling as a safety criterion in LAB selection protocols, irrespective of the functional performance of the candidate strain [31]. In contrast, the remaining strains produced tyramine at low concentrations of 30–50 μg/mL. Particularly, KS 543 showed no detectable levels of either tyramine or histamine across all tested conditions, identifying it as the most favorable candidate among the evaluated strains from a food safety perspective.

## 4. Discussion

This study highlights the importance of systematic strain screening for substrate-specific fermentation, particularly in the context of cereal processing by-product valorization. The inter-strain variability in carbohydrate and protein degradation among the 50 LAB strains confirms the strain-dependent nature of LAB enzymatic systems, consistent with previous reports describing metabolic diversity within LAB species [6,7,8]. The 2.4-fold and 2.2-fold differences observed in carbohydrate and protein degradation capacities, respectively, underscore that functional potential cannot be reliably inferred from taxonomic classification alone, indicating the importance of empirical, activity-based screening strategies when selecting candidates for defined fermentation objectives [32,33]. Such findings are consistent with genomic studies demonstrating that the number and type of cell-envelope proteinase (*CEP*) genes vary between 1 and 4 copies depending on the strain, contributing to substantial intraspecies variability in proteolytic output [23,34]. The stringent dual-threshold criteria applied in the present study, which simultaneously required both carbohydrate (≥25.25 mm) and protein degradation (≥17.98 mm) activities, represent a screening framework for identifying LAB strains with bifunctional enzymatic capacity suitable for efficient cereal substrate fermentation. Raveschot et al. reported proteolytic clear zone diameters ranging from 28 to 39 mm for *Lactobacillus helveticus* and *L. delbrueckii* strains isolated from traditional Mongolian dairy products using a skim milk agar-well diffusion assay, with a selection threshold of ≥28 mm [23]. Although the selection criteria slightly differed, the highest-performing strain in the present study exhibited a comparable proteolytic clear zone diameter of 25.45 mm. These findings suggest that the meju-derived *L. paracasei* isolates possess proteolytic activity within a range similar to that of well-characterized dairy LAB strains, highlighting their potential as efficient proteolytic agents in fermented food applications. Similarly, amylolytic *Lactobacillus fermentum* isolated from starchy substrates has been reported to produce a starch-hydrolysis halo of 45 ± 1.5 mm on MRS-starch agar after 48 h of incubation [35]. The largest carbohydrate-degradation zone observed in the present study (29.52 mm) is slightly lower than this value, which may reflect differences in screening conditions, including incubation temperature (37 °C vs. 40 °C) and inoculation method (paper disc vs. well diffusion), as well as inherent strain-specific differences in amylolytic capacity.

The predominance of *L. plantarum* and *L. paracasei* among high-performing strains aligns with their established roles in food fermentation and recognized enzymatic versatility [5,6]. Both species possess metabolically flexible genomes; *L. plantarum* in particular encodes an extensive repertoire of carbohydrate-active enzymes and proteinases, which may contribute to its performance across diverse substrates [33,36]. The origin of all selected strains from meju, a traditional Korean soybean fermentation system characterized by high protein content and diverse microbial succession [37,38,39], suggests that long-standing protein-rich fermentation ecosystems represent valuable reservoirs of LAB with robust proteolytic systems. Ecological adaptation within such niches may facilitate the selection of strains possessing well-developed cell-envelope proteinase systems and intracellular peptidase networks, contributing to efficient amino acid liberation across varied plant-based substrates [20,40].

The enhancement of BCAAs during fermentation reflects the coordinated action of the LAB proteolytic system. This process is initiated by extracellular proteinases, including CEPs in strains that harbor functional *CEP* genes, followed by peptide transport and intracellular degradation into free amino acids [20,40]. It should be noted that not all LAB strains possess functional CEPs, and the contribution of alternative secreted proteases may vary in a strain-dependent manner [20]. These oligopeptides are subsequently internalized via oligopeptide permease (Opp) systems and further degraded by a suite of endopeptidases, aminopeptidases, and dipeptidases, ultimately releasing free amino acids including BCAAs into the fermentation medium [41,42]. The resulting free BCAAs may then undergo further catabolism via branched-chain amino acid transaminase (BCAT), converting them to α-keto acids through transamination reactions, a pathway well-characterized in LAB from fermented meat and cereal systems [41,43]. However, when substrate protein availability is high and bacterial nitrogen demand is met, net BCAA accumulation occurs, as observed in pea fermentation in the present study. Aung et al. reported that solid-state fermentation of wheat bran with *Lactobacillus acidophilus* resulted in total BCAA levels of approximately 0.175 mg/g, calculated from isoleucine, leucine, and valine contents [21]. In comparison, the present study achieved higher total BCAA contents after 48 h of wheat bran fermentation, including 0.99 mg/g for KS 543 and 1.02 mg/g for KS 550, while KS 597 showed the highest value of 2.87 mg/g.

The nutritional significance of BCAA enrichment observed in the present study extends beyond fermentation efficiency to encompass broader health implications. BCAAs, particularly leucine, are well-established activators of the mTOR/p70S6K signaling axis, which governs muscle protein synthesis and helps counteract the anabolic resistance to feeding commonly observed in older adults [19,44]. Systematic reviews have further demonstrated that BCAA supplementation, especially in combination with vitamin D, improves appendicular muscle mass, grip strength, and gait speed in sarcopenic individuals [45]. In the context of plant-based diets, where protein quality and amino acid bioavailability may be limited by the amino acid profile of individual plant sources, fermentation-mediated BCAA enrichment represents a meaningful strategy for improving the nutritional value of cereal-derived ingredients [17,21]. The BCAA-enriched fermented wheat bran produced in the present study may therefore offer functional food ingredient potential for applications targeting protein adequacy in aging, physically active, or plant-based dietary contexts. Future *in vivo* studies evaluating the postprandial amino acid kinetics and muscle anabolic responses to fermented wheat bran consumption will be necessary to substantiate these health benefit claims.

Wheat bran was prioritized as the primary fermentation substrate in this study given its status as one of the most abundantly generated cereal milling by-products both domestically and globally [12]. Unlike rice bran, which is characterized by a higher lipid content and greater susceptibility to rancidity during storage, wheat bran offers a relatively stable protein-rich matrix, making it a more tractable and reproducible target for LAB-based fermentation and valorization strategies. In contrast, the more moderate BCAA response observed in wheat bran likely reflects the structural complexity and limited nutrient accessibility of this substrate. Wheat bran contains high levels of insoluble dietary fiber (40.18 g/100 g) and complex arabinoxylan-rich cell wall matrices that can physically restrict enzymatic access to embedded proteins during fermentation, thereby reducing the efficiency of microbial proteolysis [43]. The initial decline in BCAA content during early wheat bran fermentation is consistent with the preferential consumption of available free amino acids as readily accessible nitrogen and energy sources to support bacterial growth initiation, a metabolic strategy well-documented in LAB adapting to nutrient-limited cereal matrices [6,41]. In such environments, LAB utilize free amino acids through catabolic pathways including the arginine deiminase (ADI) pathway and amino acid decarboxylation reactions as supplementary mechanisms for adenosine triphosphate (ATP) generation and intracellular pH homeostasis, prior to the activation of extracellular proteolytic systems capable of sustaining de novo amino acid liberation from substrate proteins [46,47]. The partial BCAA recovery observed in the late fermentation stage suggests that cell-wall-degrading enzyme activities, potentially including amylases and xylanases expressed by selected strains, may progressively improve substrate accessibility over time [16,43].

Strain-dependent variations observed during wheat bran fermentation further demonstrate that biomass accumulation alone does not predict functional amino acid modification. The superior growth and BCAA accumulation of strains meeting both carbohydrate and protein degradation thresholds, particularly KS 568 compared to protein-only strains (KS 597, KS 574), suggest that amylolytic activity provides a critical metabolic advantage in carbohydrate-rich substrates such as wheat bran (carbohydrate content 62.77 g/100 g). Strains lacking detectable carbohydrate degradation activity may be unable to efficiently utilize the starch and arabinoxylan fractions of wheat bran as primary carbon sources, leading to reduced growth rate, attenuated acid production, and consequently lower proteolytic output [6,32]. These findings suggest that the bifunctional enzymatic profile combining both amylolytic and proteolytic activities is an important criterion for strain selection in cereal-based fermentation applications, beyond proteolytic activity alone. Differences in extracellular enzyme secretion capacity, including the expression level and substrate specificity of CEP variants (PrtP vs. PrtR), may further contribute to the observed inter-strain variability in BCAA accumulation profiles [20,40].

Biogenic amine production is considered an essential safety criterion for microbial strains intended for food fermentation applications [48]. In the present study, histamine levels detected across all isolates were far below the FDA hazard action level of 500 ppm (approximately 500 μg/mL) for histamine in foods [49], indicating a negligible risk of histamine accumulation under the tested conditions. However, the markedly elevated tyramine production observed in KS 574 (~4500 μg/mL) and KS 550 (~1700 μg/mL) represents a potential safety risk that would preclude their use as food-grade fermentation starters without additional safety validation, as tyramine concentrations exceeding the recommended range of 100–800 mg/kg (approximately equivalent to 100–800 μg/mL under the liquid fermentation conditions applied in this study) have been associated with adverse vasopressor effects in sensitive individuals [31]. Tyramine production by KS 574 reached approximately 4500 μg/mL, which is comparable to the 5486.99 μg/mL reported for the tyramine-producing strain *Lactobacillus casei* TISTR 389 [50], further supporting that biogenic amine production is a strain-dependent trait among LAB that must be considered during starter culture selection. International safety guidelines recommend systematic screening of starter culture candidates for biogenic amine production potential, including evaluation of amino acid decarboxylase activity [48]. In contrast, KS 543 produced no detectable tyramine nor histamine under the inducing conditions applied, while simultaneously demonstrating strong dual degradation activity and robust BCAA accumulation during fermentation. This combination of functional performance and favorable safety profile indicates that KS 543 is a suitable candidate for wheat bran fermentation. The absence of tryptamine, cadaverine, and spermidine production across all tested strains further supports the overall favorable safety characteristics of meju-derived LAB isolates for food fermentation purposes.

These findings support the feasibility of applying LAB-mediated fermentation to cereal processing by-products, including wheat bran, as part of broader efforts toward resource valorization and sustainable utilization of agri-food residues [15,16]. The results of the present study demonstrate that strain selection based on bifunctional enzymatic screening, combined with safety evaluation through biogenic amine profiling, provides an approach for selecting LAB strains suitable for functional food ingredient development from cereal by-products. However, the standardized liquid-state fermentation conditions and primary focus on free amino acid profiles represent limitations of the current study. Solid-state fermentation (SSF) approaches, which more closely approximate industrial wheat bran processing conditions, have been reported to yield higher BCAA enrichment and antioxidant activity compared to submerged fermentation [16,21], and should be further investigated in future studies. Broader metabolomics characterization, including bioactive peptide profiling, phenolic compound analysis, and sensory evaluation, will be necessary to fully assess the functional potential of fermented substrates for food application. From a mechanistic perspective, future transcriptomic and proteomic analyses of KS 543 and KS 595 under wheat bran fermentation conditions may provide insights into the regulatory determinants of strain-specific BCAA accumulation, particularly regarding the interplay between CEP expression, intracellular peptidase activity, and BCAA catabolism pathways [41,51].

Several limitations of the present study merit consideration, including the *in vitro* nature of biogenic amine screening and the use of liquid-state fermentation conditions, which may not fully reflect metabolic behavior under wheat bran fermentation environments. The liquid-state fermentation conditions employed (5-fold dilution) differ from solid-state fermentation (SSF), where water activity and mass transfer characteristics more closely approximate industrial processing of cereal by-products. The functional evaluation was also restricted to free amino acid profiles, and broader metabolomic characterization including organic acids, phenolic compounds, and anti-nutritional factors such as phytic acid would provide a more complete assessment of fermentation-induced substrate modification.

Notwithstanding these limitations, the present study provides a systematic and empirically grounded framework for the dual-threshold screening of LAB strains with bifunctional enzymatic capacity, and demonstrates the feasibility of wheat bran valorization through LAB-mediated fermentation. The identification of *L. paracasei* KS 543 as a strain combining robust BCAA enrichment activity with a favorable biogenic amine safety profile represents a relevant basis for the development of functionally enhanced fermented cereal by-products. Additionally, strain identification in this study relied on 16S rRNA gene sequencing, which may not provide sufficient resolution for unambiguous species-level discrimination within closely related LAB taxa such as *Lacticaseibacillus* and *Lactiplantibacillus*. Future studies employing whole-genome sequencing (WGS) or multilocus sequence typing (MLST) would provide more precise phylogenetic resolution and enable comprehensive safety genomic screening, including assessment of antibiotic resistance genes and mobile genetic elements.

## 5. Conclusions

This study demonstrates that systematic strain-level screening based on bifunctional enzymatic capacity provides a robust and practical framework for identifying LAB strains with enhanced fermentation potential in cereal-based substrates. The findings confirm that functional outcomes during wheat bran fermentation, including BCAA enrichment and biogenic amine production, are influenced by strain-specific metabolic characteristics, indicating that strain-level evaluation is important for predicting fermentation performance in defined substrate systems.

From a practical perspective, the identification of *Lacticaseibacillus paracasei* KS 543 as a strain combining robust bifunctional enzymatic activity, BCAA enrichment capacity, and a low biogenic amine profile demonstrates its strong potential for application in functional food ingredient development from wheat bran. This observation is relevant for the formulation of protein-enriched fermented cereal substrates for nutritional and functional food applications.

The integrated screening framework developed in this study, encompassing enzymatic activity evaluation, molecular identification, substrate-specific fermentation assessment, and safety profiling, provides a methodology applicable to diverse LAB collections and fermentation substrates. This approach may be used as a reference for starter culture selection in fermented food and bioprocessing applications.

Future studies should explore solid-state fermentation conditions representative of industrial wheat bran processing, alongside broader metabolomic characterization and genomic analysis of key strains, to further substantiate the functional performance and safety profiles identified in this work. Collectively, the present findings position BCAA-enriched fermented wheat bran as a promising functional food ingredient with potential health benefits relevant to protein nutrition, muscle health, and the valorization of sustainable plant-based substrates.

## Figures and Tables

**Table 1:** Molecular identification of lactic acid bacteria strains by 16S rRNA gene sequencing and BLAST analysis against the GenBank database.

Strains (KS No.)	Identification	Access Number GenBank	Sequence Identity (%)	Remarks
543	*Lacticaseibacillus paracasei*	AP012541.1	100%	Selected; met both carbohydrate and protein degradation thresholds
548	*Lacticaseibacillus paracasei*	AP012541.1	99.80%	
550	*Lactiplantibacillus plantarum*	NR_104573.1	99.80%	
568	*Lactiplantibacillus plantarum*	NR_113338.1	99.93%	
569	*Lactiplantibacillus plantarum*	NR_104573.1	99.86%	
595	*Lacticaseibacillus paracasei*	AP012541.1	100%	
545	*Lacticaseibacillus paracasei*	AP012541.1	99.86%	Not selected; met protein degradation threshold only
565	*Lactiplantibacillus plantarum*	NR_104573.1	99.93%	
574	*Lactiplantibacillus plantarum*	NR_104573.1	99.93%	
597	*Lacticaseibacillus paracasei*	AP012541.1	99.73%	

Note: Of the 50 strains screened, ten strains simultaneously satisfied both the carbohydrate degradation threshold (≥25.25 mm) and the protein degradation threshold (≥17.98 mm) and were designated as selected strains (Table 1, above line). An additional four strains fulfilled only the protein degradation criterion and were included for comparative identification purposes (Table 1, below line).

## Data Availability

The data presented in this study are available upon request from the corresponding author.

## Supplementary Materials

The following supporting information can be downloaded at: https://www.mdpi.com/article/10.3390/foods15142555/s1, Figure S1: Clear zone formation indicating carbohydrate degradation activity on starch agar plates (A) and protein degradation activity on skim milk agar plates (B) after incubation at 37 °C for 48 h; Table S1: Nutritional composition of four fermentation substrates (per 100 g); Table S2: MRM condition (All + ES + mode); Table S3: MS/MS SRM condition; Table S4: Carbohydrate and protein degradation activities of 50 lactic acid bacteria strains assessed by clear zone diameter (mm); Table S5: Changes in free amino acid contents (mg/g) during wheat bran fermentation by eight LAB strains over 48 h.

## Abbreviations

The following abbreviations are used in this manuscript:

LAB	Lactic acid bacteria
BCAA	Branched-chain amino acids
mTOR	Mechanistic target of rapamycin
CEP	Cell-envelope proteinases
KACC	Korean Agricultural Culture Collection
MRS	De Man, Rogosa and Sharpe
BCP	Bromocresol purple
PCA	Plate Count Agar
PCR	Polymerase chain reaction
PVDF	Polyvinylidene fluoride
LC-MS/MS	Liquid chromatography-tandem mass spectrometry
PTFE	polytetrafluoroethylene
HPLC	High-performance liquid chromatography
HILIC	Hydrophilic interaction liquid chromatography
ESI	Electrospray ionization
SRM	Selected reaction monitoring
UV	Ultraviolet
DDW	Double-distilled water
SD	Standard deviation
Opp	Oligopeptide permease
BCAT	Branched-chain amino acid transaminase
ADI	Arginine deiminase pathway
ATP	Adenosine triphosphate
FDA	Food and Drug Administration
SSF	Solid-state fermentation
WGS	Whole-genome sequencing
MLST	Multilocus sequence typing
