# Supplementary material for: Enhancing Nutritional and Health Benefits of Wheat Bran Through Bifunctional LAB Screening and BCAA-Enriched Fermentation
Source: Foods. 2026 Jul 20;15(14):2555. doi: 10.3390/foods15142555 (PMC13409709; doi:10.3390/foods15142555)
Supplement: Supplementary file 1 [file foods-15-02555-s001.zip › Supplement table.pdf]

Table S1. Nutritional composition of four fermentation substrates (per 100 g).

**Nutritional Components (per 100 g)**				
Component	Substrates			
	Brewed soy sauce soybean meal	Pea	Floury rice	Wheat bran
Energy (Kcal)	185.9	348.11	362.45	266.93
Sodium (mg)	3107.84	63.44	1.18	1.78
Protein (g)	22.92	22.6	8.16	15.48
Sugars (g)	-	3.33	-	3.62
Fat (g)	4.3	1.81	1.35	3.81
Trans Fat (g)	0.01	-	-	-
Saturated Fat (g)	0.72	0.33	0.44	0.58
Cholesterol (mg)	-	-	-	-
Carbohydrates (g)	21.6	71.01	81.12	62.77
Total Dietary Fiber (g)	15.44	21.31	3.41	40.18
Vitamin D (μg)	-	-	-	-
Iron (mg)	29.57	6.35	0.3	10.75
Calcium (mg)	294.03	112.98	6.72	94.82
Potassium (mg)	327.07	718.26	76.28	1,248.68

Table S2. MRM condition (All + ES + mode)

Amino acids	MRM	ConV	CID	R.T.	LOQ (nmol)
Gly	75.8 → 30.1	26	12	4.56	50
Ala	90.1 → 44.1	26	12	3.65	10
Ser	106.1 → 60.2	23	10	4.18	10
Pro	116.1 → 70.1	26	12	2.86	10
Val	118.1 → 72.05	26	12	2.21	10
Thr	120.1 → 74.0	26	12	3.09	10
Leu	132.1 → 86.15	26	8	1.64	10
Ile	132.1 → 86.15	26	8	1.8	10
Asp	134.1 → 73.95	26	12	3.08	10
Lys	147.0 → 84.1	2	12	9.93	10
Glu	148.10 → 84.10	24	16	2.52	10
Met	150.10 → 56.10	26	14	1.66	10
His	156.17 → 110.14	2	12	9.65	10
Phe	166.10 → 120.10	2	12	1.3	10
Arg	175.21 → 70.07	26	16	10.27	10
Tyr	182.10 → 136.25	2	12	1.37	10
Cys2	241.00 → 151.95	26	12	7.52	10

Table S3. MS/MS SRM condition

Amino acids	Precursor (m/z)	Product (m/z)	Collision Energy (V)	Tube lens (V)	RT (min)
Ala	89.967	43.967	9.94	33	4.56
Arg	174.967	69.967	22.3	78	7.07
Glu	147.967	83.967	15.96	63	5.64
Gly	75.883	29.967	11.09	58	4.99
His	155.967	82.967	24.62	57	6.6
Ile	132.05	68.967	17.1	52	2.47
Leu	132.05	43.967	22.76	49	2.42
Lys	147.05	83.967	16.6	71	7.56
Met	149.967	103.967	10.66	61	2.78
Phe	165.97	102.967	27.7	58	2.21
Pro	115.883	69.967	15.17	49	2.94
Ser	105.883	59.967	10.87	61	5.21
Thr	119.967	55.883	16.82	45	4.7
Val	118.05	54.967	20.75	43	3.2

Table S4. Carbohydrate and protein degradation activities of 50 lactic acid bacteria strains assessed by clear zone diameter (mm)

Carbohydrate				Protein			
**Strains**	mm	**Strains**	**mm**	**Strains**	**mm**	**Strains**	**mm**
KS 546	29.52 ± 2.83	KS 607	22.77 ± 1.82	KS 569	25.45 ± 0.24	KS 155	18.94 ± 1.30
KS 598	28.48 ± 1.43	KS 117	22.30 ± 1.13	**KS 574**	24.25 ± 1.45	KS 560	18.92 ± 0.99
KS 568	28.39 ± 1.96	KS 538	21.37 ± 1.60	KS 543	23.26 ± 0.54	KS 157	18.86 ± 1.88
KS 569	27.96 ± 3.79	KS 131	20.38 ± 1.73	KS 595	22.67 ± 0.67	KS 546	18.70 ± 0.81
KS 543	27.90 ± 2.62	KS 601	18.22 ± 2.40	**KS 565**	22.55 ± 1.03	KS 612	18.66 ± 0.98
KS 179	27.89 ± 0.57	KS 612	17.66 ± 1.64	KS 568	22.47 ± 1.26	KS 542	18.31 ± 1.59
KS 549	27.56 ± 2.43	KS 540	17.35 ± 1.77	KS 548	22.35 ± 0.59	KS 552	18.24 ± 1.54
KS 550	27.30 ± 0.97	KS 542	17.23 ± 1.59	**KS 597**	22.41 ± 2.02	KS 117	18.15 ± 0.29
KS 553	27.06 ± 2.22	KS 201	17.11 ± 9.88	**KS 545**	21.14 ± 1.40	KS 598	18.09 ± 1.51
KS 560	27.01 ± 0.47	KS 236	16.57 ± 0.45	KS 236	21.55 ± 1.98	KS 553	17.98 ± 0.80
KS 557	26.44 ± 3.25	KS 227	16.52 ± 0.34	KS 550	21.45 ± 1.12	KS 131	16.73 ± 1.11
KS 175	26.38 ± 0.26	KS 602	16.16 ± 1.01	KS 549	20.96 ± 0.43	KS 538	16.50 ± 0.88
KS 170	26.13 ± 1.22	KS 611	15.84 ± 3.00	KS 179	20.64 ± 2.12	KS 535	16.12 ± 0.95
KS 225	25.90 ± 3.62	KS 233	15.43 ± 0.65	KS 235	20.59 ± 1.79	KS 611	15.93 ± 1.36
KS 535	25.85 ± 0.57	KS 187	14.94 ± 8.98	KS 233	20.32 ± 1.25	KS 601	15.83 ± 2.02
KS 595	25.57 ± 0.19	KS 203	14.74 ± 8.51	KS 187	20.20 ± 0.27	KS 238	15.54 ± 0.78
KS 571	25.51± 0.98	KS 215	14.53± 2.62	KS 571	20.06 ± 1.06	KS 607	15.52 ± 0.92
KS 599	25.43 ± 2.80	KS 606	14.25 ± 0.64	KS 215	20.01 ± 0.73	KS 225	15.16 ± 1.87
KS 548	25.25 ± 1.87	KS 235	14.18 ± 1.14	KS 203	20.00 ± 0.36	KS 175	15.01 ± 1.41
KS 238	25.05 ± 1.07	KS 213	13.47± 1.22	KS 170	19.92 ± 2.85	KS 540	14.32 ± 0.66
KS 157	24.94 ± 2.31	KS 552	12.35 ± 7.13	KS 213	19.87 ± 0.10	KS 220	14.14 ± 1.03
KS 541	24.38 ± 3.25	**KS 574**	ND	KS 201	19.83 ± 0.57	KS 541	13.89 ± 0.38
KS 220	23.16 ± 1.29	**KS 565**	ND	KS 227	19.74 ± 1.16	KS 602	13.82 ± 0.45
KS 155	22.88 ± 2.04	**KS 597**	ND	KS 557	19.61 ± 1.32	KS 606	13.00 ± 0.54
KS 191	22.88 ± 0.61	**KS 545**	ND	KS 599	19.20 ± 0.97	KS 191	11.61 ± 0.66

Values are expressed as mean ± standard deviation of triplicate measurements (n=3). Bold strain numbers indicate strains that met the protein degradation threshold only, without fulfilling the carbohydrate degradation criterion. ND, not detected.

Table S5. Changes in free amino acid contents (mg/g) during wheat bran fermentation by eight LAB strains over 48 h.

Amino acids (mg/g)	Time (h)									
	KS 548					KS 568				
	0	12	24	36	48	0	12	24	36	48
Ala	22.90±20.20	18.05±14.71	21.21±6.35	9.53±15.68	10.15±16.29	0.30±0.07	0.31±0.10	0.30±0.07	0.31±0.03	0.34±0.05
Arg	19.32±16.43	23.60±18.57	33.68±7.93	15.52±23.58	17.57±25.98	0.90±0.06	1.09±0.06	1.22±0.05	1.35±0.03	1.47±0.01
Glu	12.43±10.56	7.12±5.58	5.66±2.13	2.24±2.98	2.48±3.30	0.58±0.08	0.61±0.05	0.55±0.00	0.59±0.01	0.63±0.01
Gly	2.88±2.59	3.04±2.22	3.43±1.13	1.37±2.66	1.36±2.66	0.63±0.17	0.82±0.15	0.57±0.21	0.86±0.36	0.44±0.34
His	1.93±0.73	1.71±0.32	1.99±0.19	1.86±0.33	2.00±0.38	1.17±0.01	1.41±0.01	1.65±0.01	1.77±0.01	1.89±0.01
Ile	4.46±0.13	0.17±0.03	0.20±0.01	0.18±0.03	0.20±0.02	0.36±0.01	0.42±0.00	0.49±0.00	0.53±0.00	0.56±0.00
Leu	1.99±0.21	0.13±0.14	0.19±0.17	0.32±0.14	0.26±0.11	0.24±0.01	0.28±0.07	0.30±.0.01	0.31±0.02	0.34±0.03
Lys	2.31±0.85	2.49±0.72	2.94±0.34	2.46±0.66	2.58±0.59	1.34±0.01	1.62±0.01	1.93±0.01	2.05±0.00	2.19±0.01
Met	0.57±0.25	0.33±0.00	0.39±0.00	0.42±0.00	0.45±0.00	0.55±0.00	0.66±0.00	0.78±0.00	0.83±0.00	0.88±0.00
Phe	1.86±0.11	1.31±0.01	1.58±0.03	1.65±0.09	1.75±0.08	0.67±0.00	0.79±0.00	0.93±0.00	0.99±0.00	1.06±0.00
Pro	8.46±7.21	7.02±5.86	8.38±4.46	6.26±8.39	7.00±9.42	0.22±0.04	0.63±0.38	0.36±0.06	0.56±0.45	0.57±0.51
Ser	0.06±1.32	ND	ND	ND	ND	0.59±.0.02	0.66±0.05	0.70±0.03	0.73±0.02	0.80±0.03
Thr	1.26±1.34	0.53±0.72	0.37±0.45	ND	ND	0.68±0.03	0.79±0.01	0.87±0.01	0.92±0.02	0.99±0.01
Val	7.93±0.06	ND	ND	ND	ND	0.04±0.00	0.07±0.01	0.06±0.02	0.05±0.02	0.07±0.03

Table S5. Changes in free amino acid contents (mg/g) during wheat bran fermentation by eight LAB strains over 48 h. (Continued)

**Amino acids (mg/g)**	**Time (h)**									
	**KS 543**					**KS 550**				
	**0**	**12**	**24**	**36**	**48**	**0**	**12**	**24**	**36**	**48**
Ala	0.28±0.05	0.36±0.03	0.27±0.01	0.45±0.08	0.39±0.04	0.48±0.15	0.50±0.07	0.52±0.10	0.38±0.12	0.42±0.11
Arg	0.89±0.05	1.15±0.11	1.25±0.09	1.18±0.02	1.62±0.22	1.14±0.03	1.27±0.07	1.44±0.04	1.31±0.14	1.23±0.02
Glu	0.56±0.06	0.60±0.05	0.59±0.01	0.68±0.03	0.70±0.03	0.84±0.06	0.91±0.11	0.72±0.03	0.66±0.09	0.67±0.05
Gly	0.40±0.17	0.89±0.22	0.72±0.48	1.21±0.80	1.08±0.59	1.57±0.15	1.83±0.13	1.41±0.19	2.12±0.68	2.17±0.34
His	1.17±0.01	1.42±0.01	1.65±0.01	1.80±0.03	1.89±0.01	1.22±0.01	1.45±0.01	1.72±0.03	1.82±0.02	1.94±0.02
Ile	0.36±0.01	0.42±0.00	0.49±0.00	0.53±0.00	0.56±0.00	0.38±0.00	0.46±0.00	0.50±0.00	0.53±0.00	0.57±0.00
Leu	0.23±0.01	0.26±0.01	0.35±0.05	0.32±0.02	0.34±0.01	0.21±0.01	0.27±0.04	0.29±0.02	0.31±0.01	0.35±0.01
Lys	1.34±0.01	1.61±0.01	1.90±0.00	2.06±0.03	2.19±0.02	1.35±0.00	1.62±0.01	1.91±0.01	2.05±0.02	2.21±0.02
Met	0.57±0.01	0.73±0.00	0.88±0.00	0.66±0.00	0.69±0.01	0.55±0.00	0.66±0.00	0.78±0.00	0.83±0.00	0.89±0.00
Phe	0.66±0.00	0.78±0.00	0.92±0.00	0.98±0.00	1.05±0.00	0.69±0.00	0.82±0.01	0.93±0.00	0.98±0.00	1.05±0.00
Pro	1.31±0.17	1.78±0.22	0.88±0.22	2.41±0.12	2.02±0.98	0.31±0.17	0.32±0.05	0.43±0.37	0.51±0.39	0.26±0.13
Ser	0.50±0.01	0.67±0.06	0.69±0.02	0.84±0.11	0.80±0.04	0.63±0.04	0.84±0.05	0.79±0.08	0.83±0.05	0.98±0.12
Thr	0.63±0.01	0.78±0.04	0.88±0.33	0.96±0.05	1.12±0.12	0.71±0.01	0.82±0.02	0.95±0.06	1.00±0.03	1.05±0.02
Val	0.06±0.02	0.03±0.00	0.04±0.01	0.05±0.04	0.09±0.02	0.12±0.07	0.18±0.12	0.11±0.05	0.09±0.09	0.10±0.08

Table S5. Changes in free amino acid contents (mg/g) during wheat bran fermentation by eight LAB strains over 48 h. (Continued)

**Amino acids (mg/g)**	**Time (h)**									
	**KS 545**					**KS 597**				
	**0**	**12**	**24**	**36**	**48**	**0**	**12**	**24**	**36**	**48**
Ala	0.37±0.05	0.39±0.04	0.41±0.04	0.44±0.07	0.54±0.06	0.97±0.12	1.59±0.18	1.40±0.40	1.85±0.16	1.85±0.24
Arg	0.90±0.04	1.06±0.15	1.18±0.06	1.25±0.05	1.44±0.15	1.21±0.21	1.86±0.21	1.22±0.02	1.98±0.13	1.95±0.38
Glu	0.64±0.05	0.59±0.04	0.61±0.02	0.63±0.01	0.70±0.04	0.68±0.08	1.04±0.04	0.79±0.00	1.21±0.07	1.22±0.21
Gly	0.64±0.39	0.65±0.41	0.71±0.16	0.67±0.36	1.25±0.23	1.28±0.54	1.89±0.23	1.21±0.62	1.12±0.34	1.41±1.01
His	1.21±0.01	1.45±0.01	1.72±0.01	1.82±0.00	1.96±0.02	1.15±0.00	1.42±0.02	1.65±0.01	1.76±0.02	1.88±0.02
Ile	0.37±0.01	0.42±0.00	0.49±0.00	0.53±0.00	0.56±0.00	0.23±0.01	0.34±0.01	0.33±0.00	0.42±0.02	0.43±0.05
Leu	0.23±0.01	0.25±0.01	0.29±0.01	0.32±0.00	0.35±0.02	1.08±0.02	1.25±0.05	1.48±0.07	1.61±0.08	1.68±0.11
Lys	1.39±0.01	1.65±0.01	1.96±0.00	2.10±0.01	2.26±0.03	1.68±0.01	2.04±0.02	2.35±0.00	2.54±0.01	2.72±0.02
Met	0.55±0.00	0.66±0.00	0.78±0.00	0.83±0.00	0.88±0.00	0.27±0.00	0.33±0.00	0.39±0.00	0.42±0.00	0.45±0.00
Phe	0.66±0.00	0.78±0.00	0.92±0.00	0.98±0.00	1.05±0.00	0.32±0.00	0.40±0.00	0.45±0.00	0.50±0.01	0.53±0.01
Pro	0.62±0.17	0.98±0.56	1.05±0.53	0.46±0.82	1.16±0.00	0.38±0.00	0.53±0.03	0.49±0.00	0.61±0.02	0.58±0.00
Ser	0.43±0.04	0.48±0.04	0.60±0.03	0.63±0.02	0.69±0.07	0.43±0.02	0.52±0.03	0.60±0.02	0.67±0.04	0.69±0.05
Thr	0.65±0.02	0.77±0.02	0.88±0.01	0.94±0.03	1.05±0.08	0.84±0.02	1.03±0.01	1.18±0.03	1.29±0.02	1.36±0.05
Val	0.10±0.05	0.09±0.04	0.06±0.01	0.08±0.02	0.11±0.02	0.40±0.01	0.64±0.06	0.54±0.00	0.75±0.07	0.76±0.12

Table S5. Changes in free amino acid contents (mg/g) during wheat bran fermentation by eight LAB strains over 48 h. (Continued)

Amino acids (mg/g)	Time (h)									
	KS 565					KS 574				
	0	12	24	36	48	0	12	24	36	48
Ala	0.36±0.01	0.33±0.00	0.37±0.03	0.37±0.00	0.55±0.05	0.28±0.08	0.34±0.08	0.32±0.03	0.47±0.14	0.46±0.01
Arg	1.03±0.14	1.20±0.12	1.15±0.12	1.33±0.20	1.62±0.12	0.99±0.13	0.90±0.00	1.06±0.00	1.13±0.00	1.20±0.00
Glu	0.82±0.18	0.78±0.09	0.64±0.05	0.72±0.08	0.85±0.04	0.67±0.14	0.82±0.18	0.83±0.04	1.14±0.28	1.02±0.08
Gly	1.22±0.34	1.39±0.33	1.20±0.39	1.45±0.45	1.55±0.12	0.68±0.26	0.59±0.32	1.03±0.19	0.82±0.23	1.16±0.75
His	1.15±0.01	1.39±0.01	1.62±0.00	1.73±0.01	1.85±0.01	1.19±0.03	1.43±0.03	1.67±0.01	1.80±0.05	1.90±0.01
Ile	0.37±0.01	0.42±0.00	0.50±0.00	0.53±0.00	0.56±0.01	0.37±0.01	0.44±0.01	0.51±0.01	0.56±0.02	0.59±0.00
Leu	0.23±0.02	0.24±0.01	0.30±0.00	0.33±0.03	0.36±0.03	0.21±0.01	0.28±.0.04	0.34±0.03	0.38±0.06	0.37±0.01
Lys	1.33±0.01	1.60±0.00	1.89±0.01	2.00±0.01	2.16±0.02	1.35±0.01	1.62±0.01	1.91±0.00	2.04±0.02	2.17±0.01
Met	0.55±0.00	0.66±0.00	0.78±0.00	0.83±0.00	0.88±0.00	0.55±0.00	0.66±0.00	0.78±0.00	0.83±0.00	0.88±0.00
Phe	0.67±0.01	0.78±0.00	0.92±0.00	0.98±0.00	1.05±0.00	0.67±0.01	0.80±0.01	0.94±0.00	1.02±0.03	1.07±0.00
Pro	0.81±0.36	0.42±0.13	0.26±0.15	0.63±0.56	0.92±0.10	0.50±0.36	1.09±0.73	1.06±0.45	1.79±0.78	1.79±0.59
Ser	0.27±0.05	0.28±0.01	0.31±0.01	0.32±0.00	0.35±0.01	0.55±0.06	0.68±0.13	0.71±0.03	0.81±0.08	0.86±0.05
Thr	0.65±0.03	0.99±0.10	1.02±0.12	1.08±0.13	1.24±0.09	0.64±0.03	0.78±0.04	0.89±0.03	0.95±0.05	1.07±0.06
Val	ND	ND	ND	ND	ND	0.07±0.02	0.12±0.10	0.04±0.01	0.16±0.13	0.11±0.05

Data are expressed as mean ± SD (n=3). ND, not detected.

Abbreviations: Ala, alanine; Arg, arginine; Glu, glutamic acid; Gly, glycine; His, histidine; Ile, isoleucine; Leu, leucine; Lys, lysine; Met, methionine; Phe, phenylalanine; Pro, proline; Ser, serine; Thr, threonine; Val, valine.
