# Supplementary figures and images for: Enhancing Nutritional and Health Benefits of Wheat Bran Through Bifunctional LAB Screening and BCAA-Enriched Fermentation
Source: Foods. 2026 Jul 20;15(14):2555. doi: 10.3390/foods15142555 (PMC13409709; doi:10.3390/foods15142555)

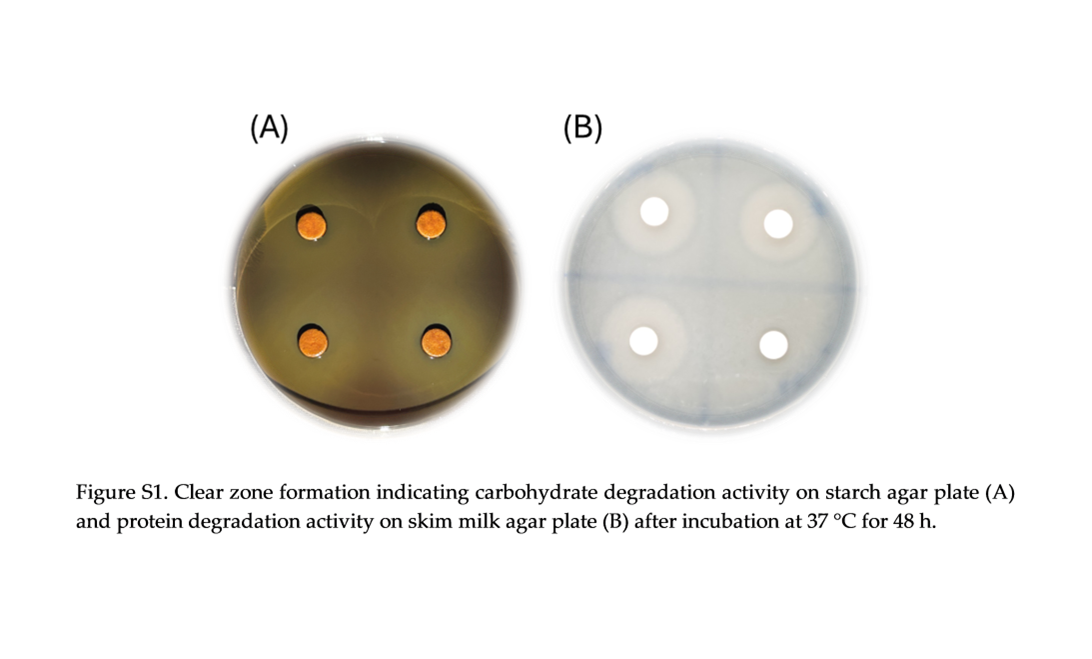

Supplement: Supplementary file 1 [file foods-15-02555-s001.zip › Supplement figure s1.png]
